# Pharmaceutical Removal with Photocatalytically Active Nanocomposite Membranes

**DOI:** 10.3390/membranes14110239

**Published:** 2024-11-13

**Authors:** Marin Popović, Silvia Morović, Marin Kovačić, Krešimir Košutić

**Affiliations:** 1 Department of Safety and Protection, Karlovac University of Applied Sciences, Trg Josipa Juraja Strossmayera 9, HR-47000 Karlovac, Croatia; 2 Department of Physical Chemistry, Faculty of Chemical Engineering and Technology, University of Zagreb, Marulićev Trg 19, HR-10000 Zagreb, Croatia; smorovic@fkit.unizg.hr; 3 Department of Polymer Engineering and Organic Chemical Technology, Faculty of Chemical Engineering and Technology, University of Zagreb, Marulićev Trg 19, HR-10000 Zagreb, Croatia; mkovacic@fkit.unizg.hr

**Keywords:** membrane technologies, nanocomposite, pharmaceuticals, photocatalysis, photocatalytic nanocomposite membrane

## Abstract

The advancement of pharmaceutical science has resulted in the development of numerous tailor-made compounds, i.e., pharmaceuticals, tuned for specific drug targets. These compounds are often characterized by their low biodegradability and are commonly excreted to a certain extent unchanged from the human body. Due to their low biodegradability, these compounds represent a significant challenge to wastewater treatment plants. Often, these compounds end up in effluents in the environment. With the advancement of membrane technologies and advanced oxidation processes, photocatalysis in particular, a synergistic approach between the two was recognized and embraced. These hybrid advanced water treatment processes are the focus of this review, specifically the removal of pharmaceuticals from water using a combination of a photocatalyst and pressure membrane process, such as reverse osmosis or nanofiltration employing photocatalytic nanocomposite membranes.

## 1. Introduction

Pharmaceuticals and personal care products (PPCPs) are a subgroup of organic compounds in the classification of emerging contaminants (EC) [1]. Organic chemicals in the environment are commonly recognised as micropollutants; i.e., they are present in the environment and potentially in drinking water in minute concentrations. Long-term exposure to micropollutants can pose serious and chronic health risks to humans and other living organisms, in particular aquatic organisms [2]. Among micropollutants, antibiotics are considered to be pseudo-persistent in the environment. This means that the concentration of all antibiotics that are released into the environment is higher than their elimination concentration from the environment [3,4]. This usually ranges between ng/L to μg/L levels, and it has been detected in all water habitats and potable water [5]. The sources of antibiotics in the environment are effluents from wastewater treatment plants and the pharmaceutical industry, sludge and manure from domestic animal farms, activated sludge from wastewater treatment plants that is used as a fertilizer and accidental spills [6,7,8,9]. Antibiotics primarily accumulate in the marine environment [9]. Most antibiotics do not get fully metabolized by the human and animal metabolisms and are excreted into wastewater [9,10]. The biological activity of antibiotics hinders their removal in wastewater treatment plants, particularly in conventional activated sludge and other biological oxidation processes. Additionally, their varied structures and chemical properties significantly impact the effectiveness of primary and conventional tertiary treatment stages as well. They can be removed partially, which is evident in their influent–effluent ratio [11,12,13]. Once in the environment, they can cause disturbance in natural biota [14,15,16] and the development of strains of bacteria resistant to antibiotics [17,18,19]. Those pharmaceuticals can be transferred via bioaccumulation in aquatic insects from aquatic to terrestrial ecosystems, which opens a new method of exposure to organisms higher on the food chain [20,21]. Table 1 contains a list of abbreviations used in this review.

## 2. Wastewater Treatment Process

Wastewater refers to water that has undergone alterations in its physical, chemical or biological characteristics due to the addition of specific substances, rendering it unfit for certain uses, like consumption. Wastewater can be divided into surface runoff water, industrial wastewater and household wastewater. Their treatment depends on the type of wastewater. Thus, industrial wastewater from certain industries must be treated due to the presence of industry-specific pollutants at high concentrations before being mixed with other wastewater. All water pollution can be categorized into three types: physical, chemical and biological. Therefore, there are three types of water treatment: physical, chemical and biological [13,22].

The wastewater treatment process turns wastewater into water that does not pose a risk to the environment or health and can be reused. This process is carried out in water treatment plants that differ in the type of water they treat. Thus, for example, sewage water treatment consists of preliminary, primary, secondary and tertiary treatment [23]. In the preliminary treatment, coarse and granular suspended material is removed. This step is necessary because floating wood, rags, paper and plastic in the wastewater would damage the equipment used in the next steps. In this step, 60% to 80% of solid materials are removed from wastewater. Greases and oils are also partially removed in this step with the use of floatation units and skimming tanks. In the primary treatment, precipitated organic and inorganic salts are removed by sedimentation in sedimentation tanks in the form of sludge. In this step, 50% to 70% of the total suspended solid and 65% of grease and oil are removed. In the secondary treatment, the remains of inorganic and organic compounds are removed. Biodegradable, dissolved and colloidal suspended organic matter is decomposed with the use of microorganisms. The most commonly used secondary treatment method is the activated sludge process. Other methods are contact beds, intermittent sand filters, trickling filters, anaerobic sludge blanket reactors and others. In tertiary treatment, specific components of wastewater that could not be removed by previous treatments are removed. Advanced oxidation processes (AOPs), advanced filtration techniques and membrane techniques as well as their combination are used here. This is followed by disinfection with chlorine, ozone or UV light. Water treated this way can be discharged into the environment or sent for further processing to meet drinking water standards [24,25,26,27,28,29].

## 3. Membrane Technology

A membrane is a thin film barrier that is permeable or semi-permeable and serves to separate fluid flow into two streams, retentate and permeate. The fluid flow occurs due to various driving forces such as the concentration gradient, pressure, electrical potential, temperature, etc. [30]. The efficiency of a membrane in separating substances is influenced by both permeability and selectivity. Selectivity refers to a membrane’s capacity to separate two different species [31,32]. The rate of water flow, also known as water permeability or water flux, increases in direct proportion to the thickness of the membrane. In contrast, solute flux is unaffected by both pressure and membrane thickness [33,34].

The membranes act as barriers, permitting the passage of certain substances while rejecting others. In the case of wastewater purification, water without pollutants can only pass the membrane. This phenomenon is achieved through different mechanisms, size exclusion and a combination of size exclusion and interaction of membrane and solute. Microfiltration (MF) and ultrafiltration (UF) membranes hinge on a size exclusion mechanism in which the pores selectively permit the passage of molecules of a certain size, while the processes of nanofiltration (NF) and reverse osmosis (RO) are based on mechanisms of charge exclusion, size exclusion, interaction of the membrane and solute and their combination [35,36,37].

Typically, membranes are fabricated from polymers such as polyethersulfone (PES), polyvinylidene fluoride (PVDF), polysulfone (PSF) or ceramics materials such as silica or alumina clay materials. The incorporation of nanomaterials into traditional membranes produces composite membranes. They can have improved permeation and separation performance compared to non-composite membranes [38,39]. Membranes can also be prepared from natural polymers like cellulose, chitin, starch or alginate [40]. They can also be prepared from inorganic ceramic materials [41].

Membrane processes are divided based on the size of the solute they retain. Applying MF, pollutants with diameters ranging from 0.1 to 10 μm can be separated. Usually, those are bacteria. MF is also used to remove oils from water when oil concentrations in water are low. UF is used to separate particles of 0.001 to 0.1 μm [42,43,44]. UF membranes retain practically 100% macromolecules, colloids and smaller pathogens like viruses from water, but they cannot be used to separate dissolved ionic species from water [45]. The NF process can separate particles with the size of 0.0005–0.001 μm [46]. It is used to separate inorganic molecules, relatively small organic molecules and divalent ions. The rejection of some solutes, with the NF process, can be more than 99%. NF is used in water softening, water purification, various industries and the production of potable water [42,47].

In the RO process, special membranes are used, which have the ability to reject all dissolved substances from water and allow only water molecules to pass through. However, RO is more energy-demanding than other pressure membrane methods. This energy is utilized to drive water molecules through the membrane against a high osmotic pressure [48]. The movement of water through RO membranes is controlled by the pore flow, not by a diffusion process in the solution, as it has been considered for decades. RO most effectively rejects all substances present in the solution (practically 99%) while allowing the water molecules themselves to pass through. The rejection of salts (ions) and organic solutes is based on the mechanisms of size exclusion, charge exclusion (with a smaller contribution) and interactions in the membrane–solution system, which depend on the compatibility of the physicochemical characteristics of the dissolved substance and the chemistry of RO membrane [49,50].

Membrane processes have two major negative aspects. The first is the fouling phenomena [51]. It causes deterioration of membrane performance, which is manifested in flux decline and lower solute retention. Fouling shortens the working lifetime of a membrane. Membranes need to be cleaned more often, and additional maintenance is required. All these reasons increase the operating costs of a process [52,53]. The fouling phenomenon and its mitigation are among the most dominant research topics in membrane technologies [54]. The second negative aspect of membrane processes is the phenomenon of concentration polarization, and it especially concerns RO and NF and less UF. This phenomenon results in the use of a higher osmotic pressure required for successful separation. With this phenomenon, the retention of substances with low molecular weight and salts is reduced with the usage of higher operating pressure [55].

Beneficial features such as superior separation performance, non-invasive characteristics, eco-friendliness and affordability outweigh the considerations against employing membrane technologies in the treatment of wastewater [55].

## 4. Advanced Oxidation Process

Advanced oxidation processes (AOPs) are a set of oxidation processes used in drinking and wastewater treatment. In 1987, William H. Glaze et al. defined these processes as “water treatment processes, which take place at temperatures and pressures close to room values, which include the formation of hydroxyl radicals in a sufficient amount to purify water” [56,57]. Since then, many AOPs have been developed (some of which do not use hydroxyl radicals but sulphate or chloride radicals), the most famous of which are ozonolysis with UV radiation (UV/O_3_), generation of hydroxyl radicals with UV radiation (UV/ H_2_O_2_), photocatalysis, the Fenton process and advanced oxidation processes based on Fenton process, supercritical water oxidation, electrochemical oxidation processes and others. Among the various methods mentioned, those involving the creation of hydroxyl radical are considered the best option for eliminating contaminants and pollutants from water [57].

In AOPs, the gradual oxidation of pollutants by radical species ideally leads to complete mineralization, i.e., the formation of CO_2_, H_2_O and inorganic ions. This is why AOPs are also called cold combustion [57]. AOPs are non-selective, so they can break down various pollutants such as hydrocarbons from oil, aromatic hydrocarbons, chlorinated hydrocarbons, paints, pharmaceuticals, explosives, compounds that give water taste and smell, compounds used in personal hygiene, bacteria and other microbes. In industrial water treatment, AOPs are used in synergy with other biological, chemical and physical water treatment processes. Potable and wastewater treatment differs according to the desired treatment goal. Thus, in the treatment of wastewater, the aim is to obtain the greatest possible biodegradability of pollutants, i.e., not to reach complete mineralization, while in the case of potable water, high standards related to microbiological contamination and more thorough removal of unwanted substances in water from wastewater favour a higher extent of mineralization [23].

Some AOPs (like ozonation) can be used before the tertiary wastewater treatment for disinfection purposes. They can be applied in primary treatment or in transitional periods between primary and secondary treatment [58].

AOPs typically involve three stages: The first is the formation of a strong oxidizing species (e.g., hydroxyl radical, superoxide radical anion, singlet oxygen) using various forms of energy, chemical, radiation, electrical or mechanical energy [59]. The second part is the non-selective reaction of the formed oxidant with pollutants in the water, whereby transformation products are formed. The third step is further oxidation of the transformation products, which leads to complete mineralization into water, carbon dioxide and inorganic salts [60].

Which transformation products are formed depends on the type of used process (type of radiation and compound that produces radicals) [61]. Transformation products undergo complex reactions, ranging from further decomposition or reacting with compounds found in the matrix or can even persist since they resist further oxidation. Such compounds can retain the effects of the compounds from which they were created and can also show more harmful effects than the parent compound [62,63,64].

Even though they are present in small concentrations, transformation products can be not only a threat to the health of people and living organisms but also a threat to ecosystems. Therefore, after AOPs, toxicity assays are carried out [65]. The simplest toxicity assay is the Microtox^®^ test. The Microtox^®^ test measures the reduction in bioluminescence in the marine bacterium *Vibrio fischeri*. The result of the inhibition of bioluminescence is interpreted by toxic substances in the solution. It is a fast, easy and reproducible method and is often used in science, but it is insufficiently sensitive at low concentrations. The assay takes 30 min [66]. Other well-known assays are the 48-h immobilization of *Daphnia magna* [67,68], 72-h growth inhibition assays of the green alga *Pseudokrichneriella subcapitata* and the 48-h growth inhibition assays of the planktonic rotifer *Brachionus calyciflorus* [69]. *V. fisheri* and *D. magna* assays are considered acute toxic assays [69]. All assays are based on the half-maximal effective concentration (EC_50_), through which the division into toxicity classes is made [70].

There are two problems with toxicity assays. The first is that they assess toxicity to different species of living creatures and kingdoms altogether; hence, something that is toxic to bacteria may not be toxic to humans and vice versa [71]. To complicate matters further, the toxicity of the treated solution depends on synergistic and antagonistic effects occurring between a plethora of transformation products and water matrix constituents. Thus, it is known whether the solution is toxic, but it is not known which component of the solution causes toxicity [66,71]. For this reason, in silico toxicity assessment methods paired with mass spectroscopy analyses could provide near real-time assessment of the treated wastewater by AOPs [72].

The advantages of AOPs are the high speed of reactions, the reduction in toxicity in many cases, the possible complete mineralization of pollutants, non-selectivity that enables the decomposition of various pollutants concurrently, no formation of sludge and the possibilities of combinations between AOPs. Their disadvantages are sensitivity to interfering compounds and the formation of oxidation byproducts and harmful inorganic compounds. In addition, the probability of the formation of toxic intermediates should also be taken into account. Some AOPs also have high running/operational costs [73,74,75].

## 5. Photocatalysis

**Figure 1:**
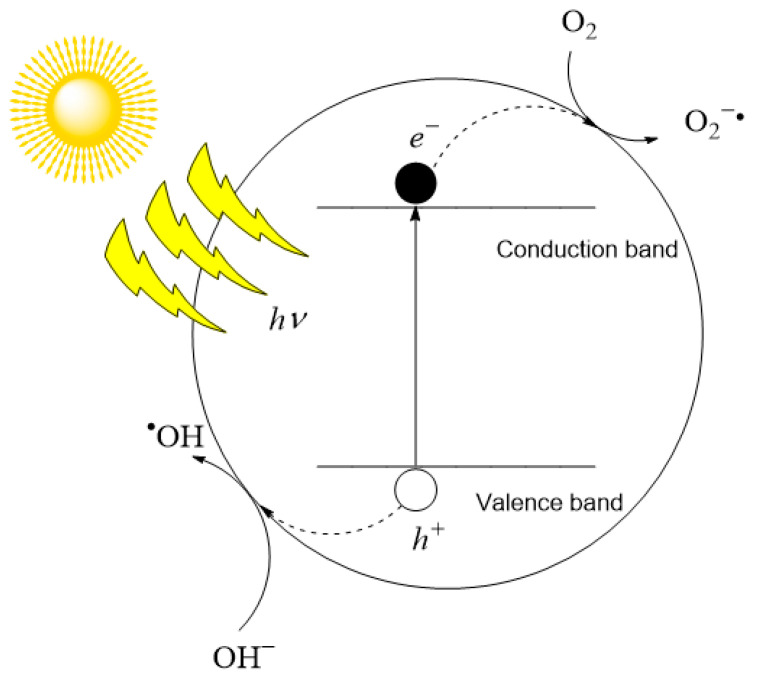
Simple photocatalysis mechanism.

Photocatalysis uses homogeneous or heterogeneous light-activated catalysts to generate highly reactive species, primarily the hydroxyl radical, for the degradation of pollutants. There are two forms of photocatalysis: heterogeneous and homogeneous. Heterogeneous photocatalysis is the type of photocatalysis mostly used in AOPs. A photocatalyst is a material in which the creation and separation of photogenerated charges (electron (*e^−^*) and hole (*h^+^*) pair) occurs upon excitation of radiation of a certain wavelength. For the photocatalyst to function, it needs to absorb energy equal to or exceeding the band gap energy (*Eg*). The band gap energy is a type of energy threshold in a semiconductor that distinguishes the valence band energy levels from the conduction band energy levels. When an n-type semiconductor is excited by radiation of a suitable wavelength, an electron passes the threshold between bands, leaving the positive hole behind (Figure 1). This procedure occurs within the bulk of the photocatalyst material during heterogeneous photocatalysis. These photogenerated charge carriers then move to the photocatalyst’s surface where they engage in both direct and indirect reactions (the formation of radicals) [76]. For this phenomenon to be effectively utilized, the separated charges must navigate to the surface of the photocatalyst. In this journey, they need to evade recombination both inside the photocatalyst and at its surface [77]. Upon reaching the surface, electrons or holes may engage in direct reactions with adsorbed contaminants on the photocatalyst’s surface [78]. Additionally, they can take part in indirect reactions with water, leading to the formation of reactive oxygen species. The electrons interact with dissolved oxygen in the solution, generating superoxide radicals, while the holes react with hydroxyl anions to create hydroxyl radicals. These resulting radicals subsequently decompose the pollutants throughout the entire volume of the solution [77,79].

### Photocatalysts

Photocatalysts are usually metal oxides such as titanium dioxide (TiO_2_), zinc dioxide (ZnO_2_), tungsten dioxide (WO_3_), iron (II, III) oxide (Fe_3_O_4_) and others [80]. As photocatalysts, compounds with more complex structures are also used, such as perovskites (compounds of the CaTiO_3_ crystal structure) [81], MXenes (two-dimensional inorganic compounds composed of thin layers of carbides, nitrides and carbonitrides of transition metals) [82] or graphite carbon nitride (g-C_3_N_4_) [83].

**Figure 2:**
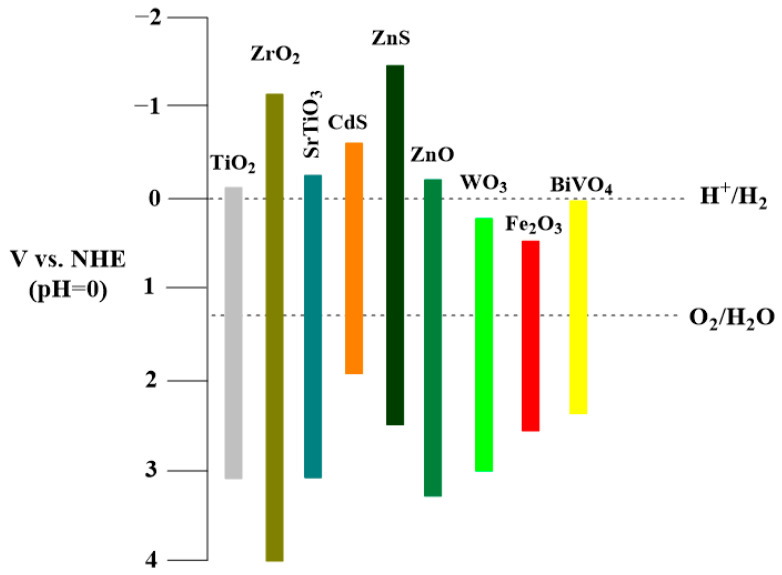
Band gaps of selected materials, with the hydrogen and oxygen evolution potentials depicted.

Metal oxides have broadband gaps due to their strongly positive valence bands, which are characterized by oxygen 2p orbitals. Specifically, their band gap widths are 2.7, 3.0–3.2 and 3.2–3.3 eV for WO_3_, TiO_2_ and ZnO, respectively (Figure 2). Such values practically eliminate their use in solar-driven photocatalysis [84].

In metal oxides, the conduction band consists of the lowest unoccupied molecular orbitals (LUMOs) of metal cations that exhibit d^0^ and d^10^ configurations, which tend to be less negative in nature. This reduction in the negative potential is not thermodynamically advantageous. To effectively reduce the width of the band gap, it is crucial to form a new valence band utilizing orbitals from other elements that do not interact with the oxygen 2p orbitals. The 6 s orbitals of Bi^3+^ bismuth, the 4d orbitals of Ag^+^ silver, the 6 s orbitals of Pb^2+^ lead and the 5 s orbitals of Sn^2+^ tin can contribute to establishing a valence band that lies above the one created by the oxygen 2p orbitals in metal oxides. Consequently, ternary metal oxides like BiVO_4_, Bi_2_WO_6_, AgNbO_3_, PbCrO_4_ and SnNb_2_O_6_ exhibit a narrower band gap and are capable of absorbing visible light [84].

The most extensively studied photocatalyst is TiO_2_, specifically the famous Aeroxide/Degussa P25. It exhibits a high level of photocatalytic activity within the 300 to 390 nm wavelength range, with the visible spectrum contributing up to 10% of its overall activity. Furthermore, it is known for its robust chemical and thermal stability, as well as appealing mechanical properties. To broaden its solar radiation absorption capabilities, the band gap energy needs to be decreased while simultaneously preventing the recombination of photogenerated charges. This is achieved through techniques such as doping and forming composites with transition metals, carbon nanotubes, conductive polymers, graphene oxide and other semiconductor materials. When developing composites, it is crucial to blend TiO_2_ with semiconductors possessing a narrow band gap, active to visible light, to create efficient composites for various photocatalytic applications [85,86].

Bismuth vanadate is an n-type semiconductor and a non-toxic yellow pigment. It is characterized by chemical stability, non-toxicity, good dispersion in solution, a narrow band gap, good corrosion resistance, good photostability [80,87,88] and excellent results in photocatalysis of organic pollutant removal under visible light [89]. It occurs in four crystal structures (polymorphs). Three of which that can be synthesized in the laboratory are monoclinic scheelite, tetragonal zirconia and tetragonal scheelite. In all crystal structures, V(V) forms a VO_4_ tetrahedra with four oxygen atoms surrounding it at the centre of the tetrahedra. Bi(III) forms a BiO8 polyhedral where Bi(III) is encircled with eight oxygen atoms. Scheelite forms and zirconia forms differ in the number of VO_4_ that surround the bismuth unit. In scheelite, the ratio of bismuth and vanadium units is 1:8, and in zirconia form, that ratio is 1:6. The local surroundings of bismuth and vanadium are more distorted in the monoclinic form compared to the tetragonal structures [77]. Of all the polymorphic structures, monoclinic scheelite shows the best photocatalytic activity, with its slander band gap of 2.4 eV and distortions in the structure that enable better charge separation. Activity under solar radiation makes it suitable for use in green technologies [90,91].

The main limitation of the monoclinic scheelite polymorph of bismuth vanadate is the bulk carrier recombination that is the result of low carrier mobility [77]. Further constraints include inefficient electron transport and hole transfer through the semiconductor–electrolyte interface. These downsides are compensated by a long diffusion length and distinctively long lifetime of photogenerated carriers, which result in high quantum effectiveness of the photocatalysts [84].

## 6. Photocatalysts Improvement

The photocatalytic activity of the photocatalyst can be improved in various ways. Some of these are doping, constructing composites, cocatalysis, synthesis of different nanostructures (changes in morphology and crystal structures) and crystal facet engineering [79].

Since modified TiO_2_ will be the most common photocatalyst in this review, it is necessary to explain the benefits of that modification. The most used modification is the formation of composites and then doping. Since there are more possible modifications of photocatalysts, we will explain them on an example of monoclinic scheelite bismuth vanadate because that photocatalyst is known to have many modifications, which are more explored than the modification of TiO_2_. Those modifications bring more photocatalytic activity to a bismuth vanadate photocatalyst than they would bring to a TiO_2_ photocatalyst, which basically has only two options for performance enhancement: doping and composite formation.

### 6.1. Doping

Doping is a frequently utilized approach to improve photocatalytic properties. This process entails the addition of one or several elements into the crystal lattice of the photocatalyst. If the added element functions as an electron donor, it is classified as n-type doping; conversely, if it serves as an electron acceptor, it is termed p-type doping. In the band gap of the photocatalyst, new energy levels are created with the contribution of the aforementioned elements, resulting in enhancement of overall photocatalytic performance. At the ideal dopant concentration, an excess of electrons or holes emerges in the conduction or valence band. The positive impacts of doping on the photocatalyst include a decrease in the band gap, heightened electrical conductivity, enhanced charge pair separation ability and improved adsorption of molecules on the photocatalyst’s surface [77]. Doping can be performed with metals or non-metals, by introducing one or more elements into the processes of photocatalyst synthesis [92].

Doping stands out from alternative techniques for enhancing photocatalysts as dopants infiltrate the crystal lattice composition, altering the element ratios within the photocatalyst’s structure. The impact of doping is evident in the modification of the band gap width and the alteration in charge transport and separation. By integrating new elements into the crystal lattice, the efficiency of electron and hole transfer from the photocatalyst’s bulk to its surface can be enhanced [77]. Cocatalysis and doping are frequently mistaken for each other. In cocatalysis, the band gap width is not directly affected, and elements or structures are placed on the photocatalyst’s surface to serve as reaction sites, leaving the photocatalyst’s structure unchanged [93].

### 6.2. Creating Composites

**Figure 3:**
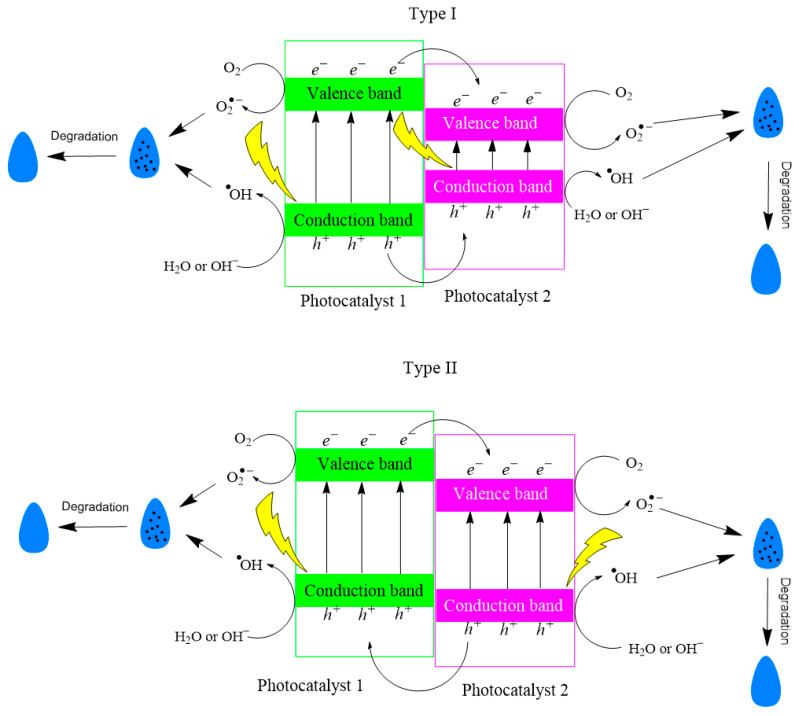

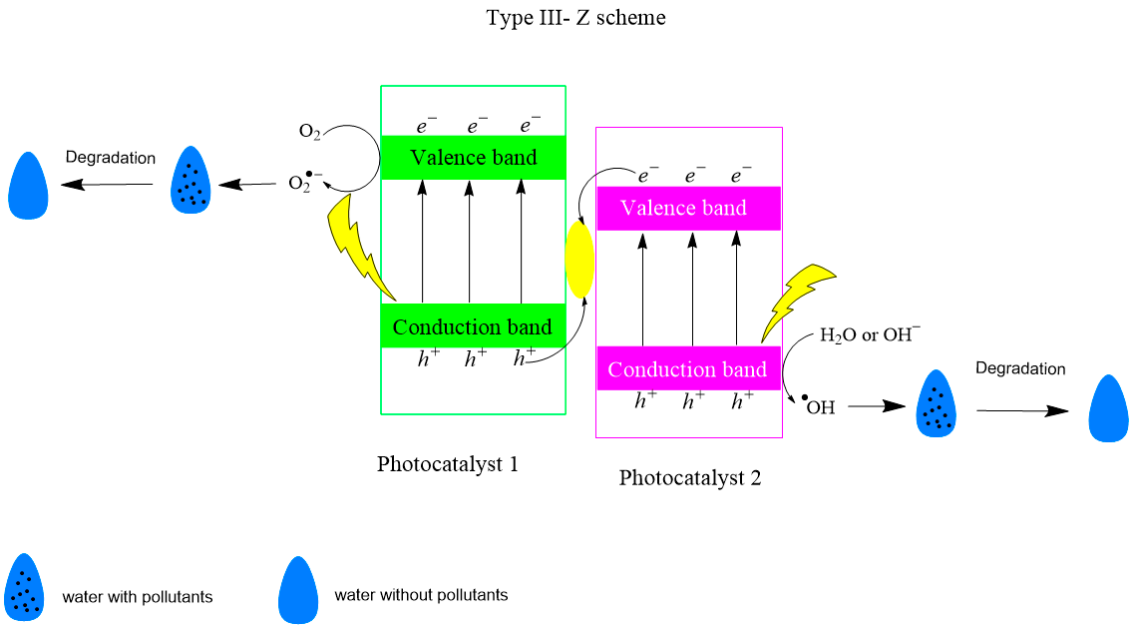
Types of composites.

Composites are compounds made from two or more different materials. If the composites are functional, they can be used for charge transfer between photocatalysts and thus reduce the influence of recombination during photocatalysis. Apart from the classic p–n type junction (used in photoelectrochemistry), composites can be divided into three types depending on the position of their band gaps, one against the other. In a type I compound, the first photocatalyst has a wider band gap (*E*g_1_) compared to the second photocatalyst (*E*g_2_), positioning *E*g_2_ within *E*g_1_. Electrons transition from the photocatalyst’s valence band with the higher energy level to the photocatalyst’s valence band with the lower energy level, while holes move in the same way between the conduction bands of both photocatalysts. Generally, this compound does not improve the photocatalytic characteristics of the composite. In a type II compound, the valence band and conduction band of the first photocatalyst are positioned at higher energy levels than their counterparts in the second photocatalyst. This leads to electron migration from the first to the second photocatalyst and hole migration in the opposite direction. Effective contact between the photocatalysts promotes this process during radiation, reducing charge carrier recombination. In a type III compound, both bands of the first photocatalyst are energetically higher than the other photocatalyst. Recombination occurs between the holes of the first photocatalyst and the electrons of the second. If a type III compound includes an electron mediator (e.g., elemental silver) between the photocatalysts, it is known as the Z-scheme and is utilized in photocatalysis applications (Figure 3). The formation of specific radicals is dependent on the opto-electronic properties of the composite material [77,85,94].

The mechanism of action of the composite on pollutants in the solution depends on the materials that enter the composition of the composite and the methods of synthesis (that is, the efficiency of the resulting compound), so composites of nominally the same composition can produce different reactive oxygen species due to differences in morphology, surface exposure, defects during synthesis and other reasons [77].

An interesting property of some composites is the homogenization of band gap energies to Fermi levels. This is an example of the BiVO_4_/TiO_2_ composite. That composite should be a type I compound because the band gap of TiO_2_ is wider than the band gap of BiVO_4_, and the band gap of TiO_2_ should surround that of BiVO_4_, but this does not happen in the compound because the positions of both bands gaps are shifted, so this becomes a type II compound. After the thermodynamic equilibrium is established, the valence and conduction bands of BiVO_4_ have a lower energy position than those bands in TiO_2_ [77].

### 6.3. Cocatalysis

A cocatalyst is a material commonly introduced on the surface of the photocatalyst [95] with the aim to improve the activity of the photocatalyst. Cocatalysts enhance light absorption, improve photogenerated charge separation, provide new active sites and enhance the stability of the photocatalyst. Cocatalysts usually have little to no photocatalytic activity of their own and are hierarchically subordinate to the photocatalyst. On the other hand, composite materials involve two or more photocatalysts, whereby each component remains in a distinct phase. In composites, photocatalytic materials perform synergistically, and the resultant activity is greater than that of individual components alone.

Photodeposition stands as one of the methods for the preparation of cocatalysts, encompassing both reduction and oxidation photodeposition techniques. In reduction photodeposition, metal ions present in the solution are deposited onto the photocatalyst’s surface. Conversely, oxidation photodeposition involves the creation of metal oxide or hydroxide nanoparticles on the photocatalyst’s surface from the solution’s metal ions. This process can be conducted at room temperature, requiring solely a reactor and a radiation source for successful particle synthesis. Theoretically, the location where the cocatalyst attaches to the photocatalyst can be controlled. By adjusting reaction parameters, such as the use of a sacrificial electron donor, radiation type and pH, the size of the photodeposited particles can be managed [93].

### 6.4. Change in Morphology and Crystal Structure

As photocatalytic reactions take place on the photocatalyst’s surface, its efficiency is linked to the presence of electrons and holes on the surface. The photocatalyst’s effectiveness is also influenced by particle size, particle structure (crystal regularity) and the photocatalyst’s morphology. In larger crystals, the photocatalytic process is often hindered due to recombination. To enhance the catalyst’s photocatalytic performance, it is advisable to use small, uniformly sized photocatalyst particles [96]. Various bismuth vanadate structures were obtained by different methods of synthesis, which mainly show photocatalytic activity [89,96,97].

The morphology of the synthesized photocatalysts can be influenced by using different surfactants and synthesis methods [98]. Examples of this are syntheses with ethylenediaminetetraacetic acid (EDTA) [99,100,101,102] and Triton X-100 [103].

Bismuth vanadate can be synthesized by various methods, the most common of which are the hydrothermal method, coprecipitation method, synthesis methods assisted by ultrasound, flame spray method, sol–gel method, synthesis methods assisted by microwaves and others [92,104,105,106,107].

An example of the formation of different morphological structures of bismuth vanadate is an example of hydrothermal synthesis with and without triblock copolymer P123 (as a surfactant) at different pHs. The structures of the resulting products vary from polyhedra, rods, tubes, spheres, hollow spheres and leaves [108]. In all of the above, control of the temperature, the length of the reaction, control of the pH and the ratio of the reactants are often used methods for the synthesis of the desired form of bismuth vanadate [96].

Zhang et al. classified the morphologies of bismuth vanadate into 10 groups: sphere-like BiVO_4_, flower-like BiVO_4_, rod-like BiVO_4_, peanut-like BiVO_4_, polyhedron-like BiVO_4_, olive-like BiVO_4_, microtube-like BiVO_4_, dumbbell-like BiVO_4_, needle-like BiVO_4_ and other morphologies. The above-mentioned morphologies mostly consisted of monoclinic bismuth vanadate and were synthesized hydrothermally with pH control and the addition of a certain surfactant. The photocatalytic activity of those materials was enough to remove dyes from solutions [96,109].

The unique form of junction, not a composite but a result of synthesis conditions, is a connection between two crystal structures of the same compound within the material. A recent investigation conducted by Yan et al. established a junction between tetragonal zirconia and monoclinic scheelite within a newly synthesized material composed entirely of bismuth vanadate. The broader band gap of tetragonal zirconia in comparison to monoclinic scheelite facilitates charge transfer, akin to a type I composite compound (Figure 3). This process reduces recombination and enhances the material’s photocatalytic performance [110]. This type of junction is called iso-type homojunction [111,112].

### 6.5. Crystal Facet Engineering

In their review paper, Chen et al. list and interpret examples of surface engineering in monoclinic bismuth vanadate. Surface engineering is based on the spatial distribution of photogenerated charges in the bismuth vanadate structure, synthesis control and structural changes during doping. The primary purpose of surface engineering in photocatalysts is to manage the charge distribution as the charges travel from the bulk of the photocatalyst to the catalyst’s surface. Since monoclinic bismuth vanadate has two types of facets, {010} and {110}, the photogenerated charges during photocatalysis travel towards one type of facet. Electrons travel to the {010} facets and holes to the {110} facets. Thus, some facets become more negative and others more positive. The exact reason for such charge separation is not known. Such charge division has its application in directional photodeposition. The next application of this phenomenon is in direct reactions on the photocatalyst, such as reactions of direct oxidation by holes, obtaining oxygen during water splitting, adsorption reactions and indirect reactions obtaining reactive oxygen species. One type of surface is always more suitable for the mentioned reactions, so the goal would be for that type of surface to be exposed, that is, to be as wide as possible, have a larger surface and be closer to the bulk of the photocatalyst to avoid recombination [113]. Such surfaces were obtained in various research studies [114,115,116]. The problem is that they were actually created by accident. Now that they are described and explained, they could theoretically be applied to design the synthesis of new forms of bismuth vanadate and improve existing materials, but these examples were synthesized under specific conditions, so their universal application is questionable. Thus, when creating a composite, the goal would be to obtain a compound of bismuth vanadate through its exposed surface, towards which the charges that participate in the interaction with other components of the composite travel, but in practice, this does not happen spontaneously [113].

It is advisable to synthesize small structures containing numerous facets with multiple edges and imperfections caused by defects in the crystal lattice. These imperfections would appear on the structure’s surface, creating highly reactive catalytic sites on the photocatalyst’s surface [113].

## 7. Catalytic Membrane Reactors

The catalytic reactor is an advanced system in which photocatalysis is coupled with membrane separation technology. These reactors can be categorized into membrane extractors, membrane distributors and membrane contactors based on the function of the membrane. They find applications in various synthetic industrial processes. In membrane extractor reactors, the membrane selectively eliminates components from the reaction mixture. The membrane distributor reactor enables the precise introduction of reactants into the reaction mixture. Conversely, the membrane contactor reactor improves the engagement between the reactants and the catalyst. Thanks to the dual-sided characteristic of the membrane, there are two methods for reactants to engage: forced flow-through and interfacial contactor. The interfacial contactor mode consists of delivering reactants from both sides of the membrane, whereas the forced flow-through mode involves supplying blended reactants from one side of the membrane in a dead-end filtration configuration [30]. In wastewater treatment, membranes have the roles of extractor (selective membranes) and contactor (use of force-flow procedures).

**Figure 4:**
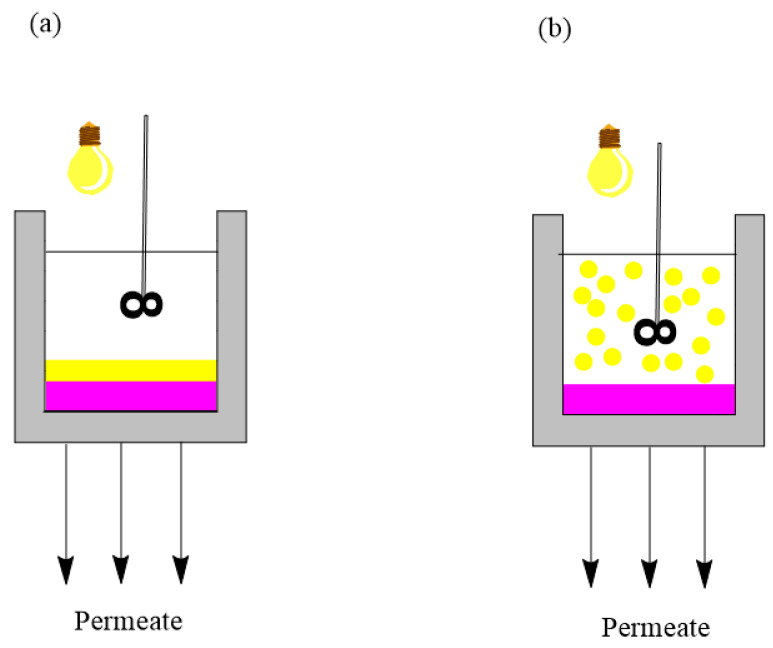
(**a**) Immobilized photocatalytic membrane reactor and (**b**) suspended photocatalytic membrane reactor.

Photocatalytic membrane reactors contain a light source, membrane and photocatalyst. They are classified based on photocatalyst loading into types: suspended photocatalytic membrane reactors and immobilized photocatalytic membrane reactors (Figure 4).

**Figure 5:**
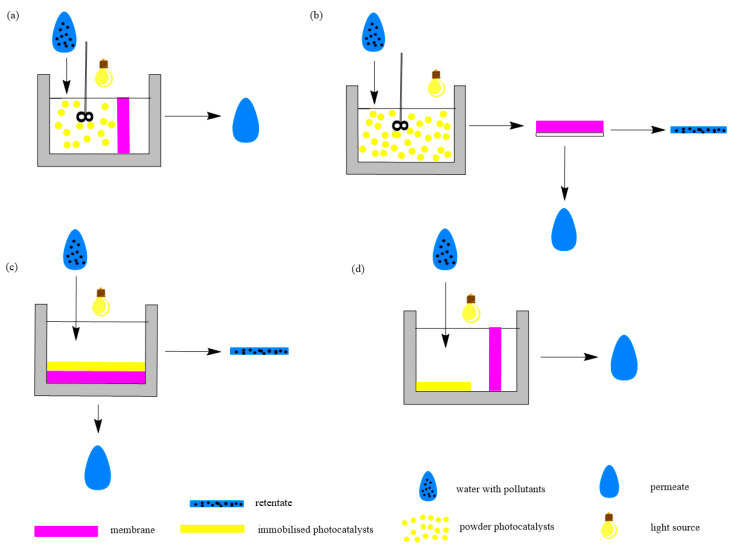
Variants of (**a**,**b**) suspended photocatalytic membrane reactor and (**c**,**d**) immobilized photocatalytic membrane reactor.

In a suspended photocatalytic membrane reactor, the photocatalyst is suspended in a solution of pollutants in a reactor (like a slurry reactor). Photocatalytic and membrane processes are separated: First, photocatalysis occurs, then membrane separation. There can also be an additional filtration step just to remove the photocatalyst from the feed and then filtrate the permeate. In a suspended photocatalytic membrane reactor, the photocatalyst can react with pollutants using its whole active surface. Those reactors are more effective than immobilized ones, but they have a pressing issue, and that is the removal of the suspended photocatalyst. There are two major variants of this reactor (Figure 5a,b). The first one is when the membrane is submerged in the slurry reactor, and the membrane filters the photocatalyst and the pollutants. The second variant is a slurry reactor followed by a membrane separation unit. In immobilized photocatalytic membrane reactors, the photocatalyst is fixed on a carrier material. Therefore, it can only react with the active surface that is not connected to the carrier material, i.e., exposed to both the treated water and illumination. Those reactors are less efficient than suspended photocatalytic membrane reactors (the reaction takes longer), but they have two major advantages: The photocatalyst is part of a membrane, so it does not need removal, and the photocatalytic membrane is easily reused. There are also two variants of this reactor (Figure 5c,d). One approach involves a photocatalytic membrane, where the photocatalyst is either embedded within the membrane’s matrix or fixed onto its surface. The other method features a submerged membrane integrated with a photocatalytic-coated reactor. In this case, the membrane and photocatalyst are distinct within the reactor, with photocatalysis taking place on the coated catalyst before membrane separation occurs [117,118].

## 8. Nanocomposite Photocatalytic Membranes

There are two most common combinations of AOPs and the membrane process. Those are a combination of ozonation and catalytic ozonation with the membrane process and a combination of photocatalysis and the membrane process [55]. In this review, the focus will be on photocatalytic nanocomposite membranes. Nanocomposites are materials where one phase contains particles within the nanometer range (10–1000 nm). In general, composites are formed by merging two or more materials that have different chemical and physical properties, which are divided by their interface. These materials exhibit specific traits that the separate components do not possess. Composites usually include a continuous phase, referred to as the matrix, along with a dispersed phase. In the context of nanocomposite photocatalytic membranes, the matrix is the membrane itself, while the dispersed phase is the photocatalyst, which may also be a composite of multiple photocatalysts. Nanocomposites can be classified based on the matrix as metal matrix nanocomposites, polymeric matrix nanocomposites, polymer/layered silicate nanocomposites and polymer/ceramic nanocomposites [119].

Nanocomposite photocatalytic membranes are engineered to reduce the negative aspects of photocatalysis and membrane technologies. Membrane fouling is the buildup of different solutes, which forms a layer on the membrane’s surface or obstructs its internal pores. This phenomenon results in diminished permeation rates, elevated transmembrane pressure and lower efficiency, ultimately leading to a reduced lifespan for the membrane. Cleaning fouled membranes demands considerable quantities of cleaning agents, leading to increased maintenance expenses. Fouling can occur on the surface or within the membrane’s internal framework [120].

The main disadvantage of photocatalysts is the incomplete mineralization of pollutants. In theory, photocatalysis should mineralize organic compounds to CO_2_, H_2_O and inorganic salts. Because pollutants have complex structures this rarely happens; instead, they get degraded into smaller organic compounds or even new toxic compounds. Those transformation products might keep the activity of the parent compound, lose the activity or have amplified activity of the parent compound. That is the reason toxicological tests are applied after the usage of AOPs.

With the combination of photocatalysis and the membrane process, these two disadvantages would be eliminated. If the fouling is caused because of an undesired structure of the molecule, the photocatalyst would degrade that molecule (with the release of radical oxygen species) into transformation products that have different structures, and some functional groups might get lost in the process. Degradation would affect the fouling process, which would despair with degraded molecules. If the fouling happens in the interior structure of the membrane, molecules that cause the fouling might get adsorbed onto the surface of the photocatalyst and then get degraded or degraded in an indirect way. This would cause the self-cleaning of the membrane. Since the pollutant molecules do not pass the membrane, the result of the process would be purified water without pollutants or their transformation products, so there is no need for toxicological tests.

Nanocomposite photocatalytic membranes are constructed with the idea that the new membrane will have better properties than the membrane without modifications. Those enhanced properties are mechanical properties, thermal stability, hydrophilicity, permeability, porosity and chemical stability [120].

The goal is to make a membrane that is more stable, has a high membrane flux, high percentage rejection of contaminants (high selectivity) alongside good antifouling properties. Integration of photocatalysts could also give membranes new properties like antimicrobial properties [121,122,123], chemical resistance, targeted degradation and magnetic properties [30].

Phase inversion and interfacial polymerization are the most used techniques to prepare polymer-based membranes. Other techniques are the colloidal precipitation method, sintering, stretching, dip coating, template leaching and electrospinning [30]. There are two main ways of preparation of a photocatalytic membrane; the first is deposition on the surface of the matrix, and the second is direct entrapment of the photocatalyst within the polymer matrix. There is also a combination of these two approaches [120].

Mixed matrix membranes are produced when the photocatalyst is entrapped within the polymer matrix. Techniques used for that purpose are spin coating, dry–wet spinning tape casting and electrospinning. Coated membranes are produced when a photocatalyst is deposited on the surface of the matrix. Techniques used for that purpose are atomic layer deposition, sputtering, electrospraying and dip coating [124,125]. Mixed matrix photocatalytic membranes have shown better results in contaminate removal than membranes coated with photocatalysts. The reason for that is a larger active surface area which allows more interactions between photocatalysts and pollutants. On the other hand, membranes coated with photocatalysts are easier to clean and have higher catalyst recovery and reuse potential [126].

**Figure 6:**
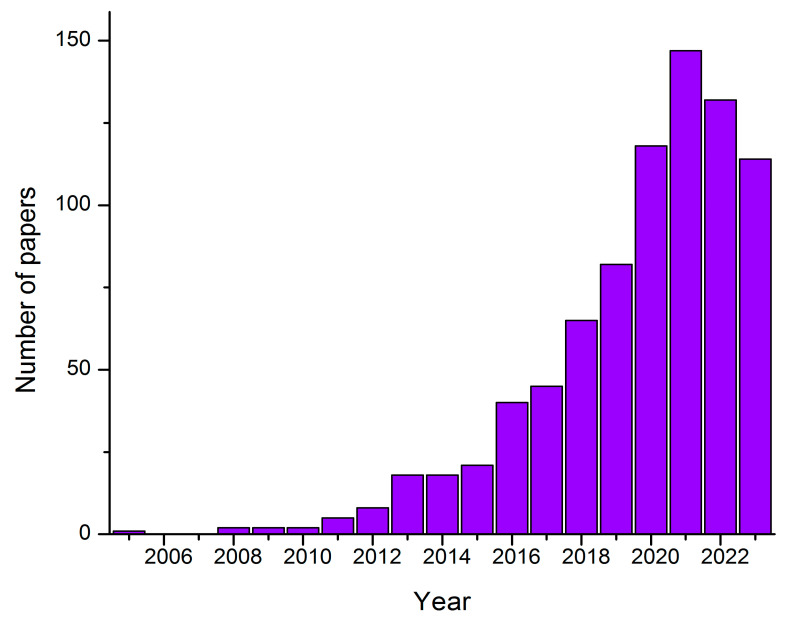
Results of literature search using the term “Photocatalytic nanocomposite membrane” (Web of Science, 6 June 2024).

The increase in the number of published papers with the keywords “photocatalytic nanocomposite membranes” used to perform a search of the scientific database Web of Science is shown in Figure 6. In the last few years, there has been a slight decline in published papers, but since 2020, they have passed the 100-paper mark annually.

Photocatalytic nanocomposite membranes are mostly used for the removal of dyes from water [123,127,128,129,130,131,132,133,134,135,136,137,138,139,140,141]. Various nanocomposite photocatalytic membranes have been used for removal: dairy wastewater treatment [142], bacteria [143,144] and viruses [144], water desalination [145], oil-water separation [146], heavy metals [147], endocrine disruptors [148] and pesticides [149].

## 9. Emerging Materials

Some materials are used to enhance photocatalysts in the nanocomposite part of a membrane; others can be photocatalytic membranes by themselves.

### 9.1. Graphene Oxide and Reduced Graphene Oxide

Graphene oxide (GO) is a two-dimensional carbon nanosheet characterized by a hexagonal honeycomb arrangement and the presence of oxygen-functionalized groups. It comprises both hydrophobic elements (the graphene part) and hydrophilic components (such as carboxyl and hydroxyl groups) [150]. This material is endowed with a variety of oxygen-based functional groups, including epoxy, carbonyl, carboxyl and hydroxyl, located on its basal plane and edges, which enhances its ability to disperse in both water and organic solvents [151,152]. GO displays amphiphilic properties, allowing it to bond with water-insoluble particles through mechanisms such as hydrophobic interactions, π–π stacking or non-covalent bonds [150]. Its remarkable flexibility enables the formation of nanochannels comprised of hydrophobic interlayers, which contain hydrophilic pores, resulting in the electrostatic repulsion of small ions and an increase in water flux [153]. The functional groups present lead to the swelling of GO membranes in aqueous environments, where water molecules create layers between the graphene oxide sheets. In water, the interlayer spacing of GO membranes can expand up to three times compared to dry conditions, permitting the passage of species with a hydrated radius of 0.45 nm or smaller, thereby diminishing the selectivity of GO membranes [152]. The reduction in graphene oxide yields reduced graphene oxide (rGO), a variant with fewer oxygen-containing functional groups, lower water dispersibility, hydrophobic characteristics and a more defective structure than graphene [150,154].

GO membranes can be categorized as free-standing, supported or nanocomposite. Free-standing membranes are created by applying a GO layer onto a filter paper or another substrate. They are very thin and prone to damage and deformation when subjected to pressure, making them the least common type of membrane. Supported membranes involve depositing a GO layer onto the surface of another membrane that can endure operational conditions [154,155]. When GO is used for its antibacterial and antibiofouling properties, GO should be the exposed part of the nanocomposite membrane [156].

Graphene oxide is also a photocatalyst [157]. The band gap of the material is influenced by the oxygen/carbon ratio and the types of functional groups within its structure. Typically, the band gap falls within the range of 2.1–2.2 eV, corresponding to a wavelength of 550 to 560 nm within the visible light spectrum. GO is commonly incorporated into composites alongside TiO_2_ and ZnO, particularly in nanocomposite membranes. In these composites, GO facilitates electron transfer to the other photocatalysts, reducing electron-hole pair recombination. It also narrows the composite band gap, enabling activity under visible light. Oxygen functional groups on a high surface area assist in guiding TiO_2_ aggregation during the synthesis of the composite, enhancing pollutant adsorption. GO-based membranes have those properties: photocatalytic degradation of fouling compounds, antibacterial properties and increased hydrophilicity [158,159,160,161].

Graphene oxide-based membranes have a couple of downsides; first is the high cost of materials, followed by limited structure stability and technological challenges in large-scale membrane manufacturing [162].

GO membranes have been used for the treatment of oily wastewater [154], rubber wastewater [163], batik (a form of textile decoration) industry wastewater [164], water desalination, dye degradation and various separation processes [150].

### 9.2. Graphitic Carbon Nitride

Graphitic carbon nitride (g-C_3_N_4_) is derived from s-triazine (C_3_N_3_) or s-heptazine (C_6_N_7_) units connected through secondary or tertiary amino groups. This 2D metal-free polymer material boasts a flat surface and a 2.7 eV band gap, rendering it active under visible light, capable of generating superoxide ions and hydroxyl radicals. Its 2D layered configuration is maintained by feeble van der Waals forces. Noteworthy attributes include thermal stability, resilience to mild acids and bases, insolubility in water and organic solvents. Despite its narrow visible light absorption, high recombination rate, distorted structure and limited specific area, modifications are necessary to enhance its photocatalytic efficacy [162,165].

Graphitic carbon nitride possesses a layered configuration with triangular nanopores that enhance the rapid permeation of small molecules, such as water, exhibiting a molecular sieve-like function. Additionally, there is a defect-rich structure that can be advantageous for membrane properties by reducing the transmission path; however, this is not beneficial for industrial applications and mass production. Membranes based on g-C_3_N_4_ demonstrate superior separation performance and self-cleaning capabilities compared to traditional membranes. These membranes can either stand alone or be integrated into a membrane structure, such as being a top layer on a supporting membrane or part of a nanocomposite within the membrane’s composition. Self-supported g-C_3_N_4_ membranes consist of g-C_3_N_4_ layers or g-C_3_N_4_ composite layers. Although these membranes have been successfully synthesized, they are often overshadowed by other groups where the primary membrane is made of a different material, with g-C_3_N_4_ serving as a support layer or part of a nanocomposite particle in what is known as g-C_3_N_4_-based membranes. Membranes with graphitic carbon nitride are used for pervaporation, desalination, oil–water separation, dye removal and in photocatalytic membranes, in microfiltration, ultrafiltration, nanofiltration, reverse osmosis [152,162,166,167,168].

### 9.3. Metal–Organic Frameworks (MOFs)

Metal–organic frameworks (MOFs) consist of an organic ligand combined with metal ions or clusters, forming crystalline materials that can exhibit porosity. The usual synthesis methods of MOFs are hydrothermal or one-pot solvothermal methods, where solvents, metal ions and organic ligands self-assemble [169]. Various series of MOFs exist, including zeolitic imidazolate frameworks (ZIFs), UiO, MIL, HKUST-1, PCN and Cu-MOFs, commonly utilized in membrane applications. ZIFs are a type of MOF with imidazole-based organic ligands and tetrahedral coordination geometry transition metals like zinc, cobalt or iridium. The UiO series, associated with the University of Oslo, features a three-dimensional microporous structure with Zr ions connected to organic ligands of terephthalic acid, forming central pore cages and smaller tetrahedral corner cages [170].

In wastewater treatment, MOFs are employed as adsorbents due to their adjustably porous nature, high surface areas and strong adsorption capacities. Some MOFs exhibit photocatalytic properties due to light harvesting and ligand–metal charge transfer. Examples include MOF-5, MIL-125 and UiO-66, used for degrading dyes [171].

Initially sensitive to water content, MOF membranes have evolved to become water-stable, suitable for water treatment applications. Various strategies exist for preparing nano MOF-based composite membranes, such as integrating them into polymer matrices during synthesis, creating layered membranes or incorporating MOF nanoparticles into polymer matrices or on membrane surfaces. These strategies leverage the unique properties of MOFs, including tuneable modularity, vast surface areas and porous structures. MOF membranes find applications in dye removal, heavy metal pollutant capture, oil-water separation and, to a lesser extent, pharmaceutical and pesticide removal [172,173,174,175,176].

### 9.4. MXenes

MXenes belong to the category of 2D layered materials named after MAX phases. Those phases form hexagonal nitrides and carbides with specific elemental constitution ternary ceramic M_n+1_AX_n_ (MAX), where n ranges from 1 to 4. In MAX, M is a d-block element from groups 3 to 7, A is an element from group 13 or 14 in the periodic table of elements, while X denotes either carbon or nitrogen. The structure of MXenes comprises distorted XM_6_ octahedra that share edges and are interleaved by single planar layers of the A-group element. These materials are produced through a process involving the etching of MAX phases. Specifically, hydrofluoric acid is used to etch out the A element from the MAX phase structure, resulting in MX multi-layered carbides, nitrides or carbonitrides with an accordion-like morphology. This method leverages the unique structure of MAX phases, where the A element does not form covalent bonds with the M and X elements but is instead embedded within the MAX phase structure [152,177,178,179,180]. Furthermore, MXenes can be synthesized mechanochemically [181]. MXenes are described with the general formula MX (M_n+1_X_n_), but functionalized MXenes have a general formula M_n+1_X_n_T_x_, where T_x_ is used to describe surface terminations such as –F, –OH, –O– [182].

While MXenes are inherently metallic, functionalized MXenes can exhibit semiconductor properties [183,184,185]. Pure MXenes are not suitable for direct use as photocatalysts since they cannot absorb light independently. However, when connected to a photocatalyst capable of light absorption, charge carriers are transferred to the MXenes, which act as efficient charge carriers due to their metallic nature [186,187,188,189]. Ti_3_C_2_T_x_ is one of the most extensively studied MXenes, displaying both semiconductor and metal-like transport characteristics. Its band gap energy can be tuned between 0.92 eV and 1.75 eV based on the functional groups present on its surface. Surface functional groups facilitate strong interactions between Ti_3_C_2_T_x_ and other semiconductors, leading to the formation of effective heterojunctions for composite applications [189]. Ti_3_C_2_T_x_ offers advantages such as high mechanical strength, a negative surface charge and hydrophilicity, making it suitable for membrane fabrication. Incorporating Ti_3_C_2_T_x_ into membranes enhances their antibacterial properties and prolongs membrane lifespan [152,190,191].

MXenes exhibit a vast specific surface area, superb electrical conductivity and tuneable layer spacing and composition. The abundance of –OH, –O and additional functional groups on the surface gives MXenes exceptional hydrophilicity, making them ideal for membrane uses. Nonetheless, MXenes also show restricted layer spacing and inadequate fouling resistance, which hinders their reusability [192].

Fabrication of MXene-based membranes involves three main methods: utilizing lamellar-structure MXenes as a skeleton material, creating mixed matrix membranes with MXenes or other nanomaterials and incorporating MXenes into a thin film layer to modify existing support membranes. MXene membranes offer longitudinal and lateral transport pathways, unlike conventional membranes that only allow one-way flow. This property is attributed to the 2D nature of MXene membranes, which feature nanochannels with pores on the outer and in-plane pores on the inner side, as well as inter-galleries between MXene layers due to their laminar structure. Embedding nanoparticles in the membrane structure can enhance separation efficiency [190,193].

Bare MXene nanosheets are prone to oxidation and tend to aggregate in an aqueous environment, but when a membrane is formed from them, it possesses structural flexibility and mechanical and chemical stability. Usually, composites of MXene are used to fabricate membranes because fabrication of membranes from bare Mxenes is complicated and has a high cost [194]. MXene-based membranes are used in the treatment of oil–water mixtures [195], but nanocomposite MXene membranes (with GO, TiO_2_, Al_2_O_3_…) have been used to remove heavy metals, dyes, bacteria, humic acid, bovine serum albumine and inorganic compound and micropollutants [193,196,197,198].

There are also composite membranes synthesized as a combination of the aforementioned materials [192,199,200,201]

## 10. Improvement of Membrane Properties Without Photocatalysis

Some of the effects from Table 2 are explored in more detail. Abadikhah et al. conclude that the rGO nanosheet structure favours solvent channelling, and TiO_2_ has hydrophilic characteristics which result in high structural stability and antifouling properties on the new Si_3_N_4_/PAN- TiO_2_@rGO membrane compared to the Si_3_N_4_/PAN membrane. Experiments were performed on (dye) solutions of Bromothymol blue and Rose bengal in ethanol [202]. Du et al. attribute the increased permeability of the ZnS and GO hybrid membranes to their hydrophilic nature and the synergistic effect between them. This synergy also effectively reduces membrane fouling compared to a pure PVDF membrane.

The ZnS/GO-PVDF membrane exhibits a slower decline in flux rate thanks to its high hydrophilicity that creates a water layer, diminishing contact between the membrane surface and pollutants. The experiments were conducted using methylene blue as a test substance [203]. Ahmad et al. separated water from a synthetic oily saline solution using a polyamide membrane with a charged titania nanosheet assembled as a thin film composite. Titania nanosheets have a large surface area and high surface hydrophilicity because of the hydroxyl (–OH) functional group present at the surface in large quantities. This can benefit the separation of oil and water because oil adsorbs on the large hydrophilic surface, and water passes through the membrane. In the comparison of modified membranes versus unmodified membranes, the alterations lead to the creation of a hydration layer. This layer boosts resistance to fouling from highly concentrated saline oily water while keeping salt rejection capabilities. Furthermore, the modified membranes display superior surface characteristics, such as a longer lifespan, surface roughness and increased hydrophilicity [206]. Vatanpour et al. fabricated a TiO_2_/carbon dot-modified PSF/PA reverse osmosis membranes. The modified membranes improved fouling resistance because of the smoother surfaces with reduced contact angles. In experiments with NaCl solution, they have shown an increase in desalination efficiency and permeability and improved chlorine resistance. The use of UV during the washing process improved flux recovery [209].

## 11. Improvement of Membrane Properties with Photocatalysis with Model Substances

In this experiment, bovine serum albumine and humic acid are used as a non-migratory fouling agent [168,211,212,213,214]. Some of the effects from Table 3 are explored in more detail.

Du et al. measured higher fluxes of hybrid ZnS/GO-PVDF membranes under sunlight than without (sunlight) photocatalytic degradation. This was an indication of BSA degradation. The successful degradation of BSA is attributed to the photochemical properties of the ZnS/GO system, which favours the recombination reduction in photoinduced electron–hole pairs with electron transfer [203]. Sisay et al. produced multiple PVDF variations that underwent modifications involving TiO_2_, carbon nanotubes and/or BiVO_4_ during the coating process for membrane fabrication. Additionally, TiO_2_ was integrated into the membrane structure. These membranes were utilized for treating simulated dairy wastewater with BSA. Membranes coated with TiO_2_ and TiO_2_ combined with carbon nanotubes displayed over 97% regeneration efficiency under UV light. When exposed to visible light, coated membranes enhanced with TiO_2_, carbon nanotubes and BiVO_4_ exhibited superior regeneration capabilities due to the activity of BiVO_4_. Blended membranes demonstrated enhanced BSA rejection and anti-fouling characteristics, attributed to pore blockage and layer interactions. The rise in surface roughness led to decreased BSA retention, while the impact of TiO_2_ concentration on flux recovery was negligible. Pristine PVDF membranes exhibited higher BSA rejection rates compared to coated and blended membranes. TiO_2_-blended membranes showcased improved anti-fouling properties, superior flux rates and comparable BSA rejection rates to pristine PVDF membranes [215]. Chen et al. fabricated SnO_2_/GO-PVDF membranes with sponge-like pores. The rejection rate of the SnO_2_/GO-PVDF membrane increased compared to the PVDF membrane. Photodegradation efficiency and kinetics toward BSA under UV light are increased for SnO_2_/GO-PVDF membranes in comparison to SnO_2_-PVDF and GO-PVDF membranes indicating composite synergy [216]. Mousa et al. fabricated TiO_2_/ZnO-PVC, ZnO-PVC and TiO_2_-PVC hybrid membranes using green synthesis methods to produce TiO_2_ nanoparticles from pomegranate extract and ZnO nanoparticles from tangerine extract. Fabricated membranes were used to remove humic acid under visible light. Mechanical properties and hydrophilicity of fabricated membranes have improved. TiO_2_/ZnO-PVC had the best photocatalytic properties and rejection rate of humic acid at 98.7% [217].

### Photocatalytic Self-Cleaning

One of the reasons for creating photocatalytic nanocomposite membranes is their self-cleaning ability. Since membrane technologies are used in the last steps of the water purification process, there are some molecules that go through the process with micropollutants like bovine serum albumine in dairy wastewater [215] or humic acid in drinking water [219]. Typically, there are six main types of fouling. Biofouling results from biofilms composed of extracellular polymeric substances and microbial cells [220]. Organic fouling is triggered by dissolved organic matter and organic colloids. Colloidal fouling is induced by colloids. Scaling occurs when insoluble salts are deposited. Concentration polarization involves the buildup of rejected solute on the membrane surface. Adsorptive fouling is the result of the adsorption of different organic compounds. Fouling may manifest on the membrane’s surface or within its pores [158,221]. The photocatalyst in the membrane is there to prevent that effect [222]. Activated by light, those molecules are destroyed or transformed into smaller molecules via direct oxidation or indirect oxidation (production of radical oxygen species). Smaller molecules (transformation products of the starting molecule) in the process may lose their functional groups, which allows them to accumulate, adsorb or deposit into or onto the membrane and stay in the feed [158]. This effect can be used when membranes are washed. Degradation is also beneficial in the water purification process. If humic acid is taken as an example, it can interact with other pollutants, impact the removal of heavy metals, produce toxic disinfection byproducts, cause fouling of the membranes [223]. For this organic compound, a combination of removal methods is the best solution.

Photocatalytic activity is linked to self-cleaning in multiple research papers [221,224,225,226].

## 12. Pharmaceutical Removal with Photocatalytic Nanocomposite Membranes

Recent examples of pharmaceutical removal photocatalytic nanocomposite membranes are shown in Table 4.

Zwane et al. fabricated PMIA -WO_3_/g-C_3_N_4_, which was used for the degradation of diclofenac in water. Modified membranes have shown better properties than unmodified PMIA. The membrane with 2% WO_3_/g-C_3_N_4_ showed the highest removal of diclofenac. Modified membranes have shown stability during 10 cycles of photodegradation. The degradation of diclofenac was also followed via total organic carbon analysis [227]. Chakachaka et al. fabricated CoFe_2_O_4_-PES membranes for the degradation of naproxen in water. Naproxen is a non-steroidal anti-inflammatory drug, and CoFe_2_O_4_ is a ferrite with low band gap energy and good photocatalytic properties.

Three photocatalytic membranes were fabricated, and all showed improvement in naproxen degradation compared with the pristine one, and the best degradation of 60% was achieved by the 1% CoFe_2_O_4_-PES membrane [228].

Li et al. created bimetallic Au_0.1_Ag_0.9_ nanoparticles on TiO_2_ nanorods within a cellulose acetate membrane (Au_0.1_Ag_0.9_/TiO_2_-CA). The photocatalyst was synthesized using the sol-immobilization technique. This membrane possesses antibacterial characteristics and can decompose tetracycline by 90% in a dynamic system [229]. Koe et al. developed a photocatalytic membrane by combining carbon quantum dots co-doped with nitrogen and sulphur (N,S-CQD) with titanium oxide (TiO_2_) in polysulfone for degrading diclofenac in water. They created three membranes with varying amounts of N,S-CQD loaded on TiO_2_. The most effective membrane for diclofenac degradation under visible light was the one with 1.5 N,S-CQDs/TiO_2_ (1.5 g of N,S-CQD loaded onto TiO_2_), achieving 62.3% removal compared to the pure TiO_2_ membrane’s 3.33% removal. Additionally, this membrane displayed enhanced hydrophilicity and the highest permeability among all tested membranes. These findings suggest that incorporating N,S-CQDs onto TiO_2_ can enhance the performance of TiO_2_ under visible light instead of UV light [230].

Presumido et al. developed photocatalytic membranes consisting of graphene/TiO_2_ -α-Al_2_O_3_. Two types of graphene-TiO_2_ composites were applied to coat α-Al_2_O_3_. The first type involved synthesizing the composite by adding TiO_2_ during graphene preparation (at concentrations of 1%, 2% and 3% TiO_2_). The second type entailed uniformly coating a thin film of TiO_2_ over a graphite film structure (in 3, 6 and 9 layers). Two solutions containing four pollutants (diclofenac, 17β-estradiol, 17α-ethinylestradiol and amoxicillin) were prepared: one with ultrapure water and the other with urban wastewater post-secondary treatment, each pollutant at a concentration of 500 μg/L. Photocatalytic experiments were conducted in two reactors. The first was a batch reactor with a UVA lamp, and the second was a lab-scale system comprising a photocatalytic membrane reactor (vertical position) equipped with four external UVA lamps, a 5 L feed tank, a thermostatic bath, a magnetic stirrer, a gear pump, a control system for pump, lights, flow rate and a back pressure regulator linked to a PC to regulate transmembrane pressure. In batch tests, the pristine membrane exhibited minimal pollutant removal. The prepared membranes surpassed the pristine one, with the second type of membrane preparation (layers of thin films) outperforming the first. Experiments on the lab-scale system with a model stock solution revealed the best-performing membranes of each type. The first type, with the highest TiO_2_ content (3%), achieved pollutant rejections of 48% for DCF, 56% for E2, 54% for EE2 and 49% for AMX. The second type, with six layers of TiO_2_, showed superior results: 53% for DCF, 65% for E2, 62% for EE2 and 45% for AMX. The slightly better performance of layer-coated membranes was attributed to improved exposure of the photocatalyst to light. The membrane with the most layers (nine) experienced flux decline, leading to reduced removal rates. E2 exhibited the highest removal rate among pollutants, while AMX showed the lowest, influenced by reaction conditions affecting chemical behaviour. Experiments with urban wastewater stock solution on the lab-scale system indicated a slight decrease in pollutant rejection values and flux reduction. The first type membrane achieved maximum rejection values of 31% for DCF, 43% for E2, 41% for EE2 and 29% for AMX, while the second type reached 34% for DCF, 49% for E2, 45% for EE2 and 31% for AMX, making it slightly more effective. The decline was attributed to existing pollutants in the wastewater matrix and pollutant adsorption on the membrane hindering light-membrane contact. In the absence of light in the wastewater matrix, there was a 45% decrease in flux rate due to membrane fouling. Toxicity screening using a zebrafish embryo bioassay revealed a significant reduction in toxicity from the mentioned pollutants in both synthetic and wastewater matrices after treatment with photocatalytic membranes compared to untreated samples [231].

Errahmani et al. prepared two membranes whose matrices consist of a PVDF/PMMA composite with TiO_2_ as a photocatalyst. The membranes were used to remove dyes Congo Red and Tartrazine and antibiotic ciprofloxacin separately from water. Congo Red and Tartrazine had a rejection rate of 99% and 81%, and ciprofloxacin had a rejection rate of 15% in 30 min under UV light. This result was explained by the dependence of the ciprofloxacine structure on the pH of the solution [232]. Aldana et al. developed nanocomposite PVDF-TiO_2_ photocatalytic membranes to eliminate venlafaxine, an antidepressant, and metoprolol, a β-blocker, from ultrapure water and municipal wastewater treatment plant effluent under UV light. The membranes achieved a 99% rejection in ultrapure water but less than 35% in the effluent. The presence of carbonate/bicarbonate ions in the effluent acted as radical scavengers, limiting pollutant removal to 60% even after their elimination [233].

You et al. prepared PTFE photocatalytic membranes that have a composite consisting of S-doped graphitic carbon nitride and N-doped TiO_2_ acting as a photocatalyst (S-g-C_3_N_4_/N–TiO_2_-PTFE membrane). This membrane was used to remove tetracycline from water with 99% efficiency. This combination of doping and composite boosted TiO_2_ photocatalytic activity [234]. Cui et al. developed photocatalytic nanocomposite membranes by creating Ti-doped bismuth oxyiodide (BiOI) coated with polydopamine (pDA) on cellulose acetate (CA) substrates (Ti/BiOI-pDA/CA). Bismuth oxyiodide, a non-toxic photocatalyst, exhibits improved photocatalytic and antibacterial properties when doped with Ti. The synthesis involved producing flower-like Ti/BiOI photocatalysts with a large surface area advantageous for adsorption and degradation processes. Polydopamine, known for its high bioadhesivity and biocompatibility, facilitated a thin layer coating on various solid surfaces under mild alkaline conditions. It served to stabilize the photocatalyst on the membrane’s surface, enhancing the coupling between the photocatalyst and the membrane. This membrane effectively removed tetracycline from water, demonstrating a high photocatalytic activity of 98%, a removal efficiency of 91%, outstanding antifouling performance, as well as remarkable stability and reusability [235].

Aoudjit et al. prepared a 10 wt.% TiO_2_-PVDF–TrFE nanocomposite membrane which was used to remove niflumic acid. Niflumic acid is a non-biodegradable anti-inflammatory drug which exhibits resistance to chemical and biological degradation. Experiments were performed in a photocatalytic membrane reactor for six hours under direct sunlight exposure and under simulated sunlight exposure. The influence of pH, concentration of pollutant and source and time of exposure were examined. The best niflumic acid degradation efficiency was for 10 mg/L niflumic acid solution under direct sunlight for 6 h at a neutral pH, and it was 91%. Additionally, an artificial neural network model was developed, and the level of agreement between the actual and forecasted data, as indicated by R^2^, was 0.98 [242]. Satulu et al. developed a nanocomposite membrane comprising cellulose acetate (CA) blended with varying amounts of TiO_2_-decorated graphene oxide (GO) (0.5 wt.%–2 wt.% GO), positioned between two thin films resembling polytetrafluoroethylene (PTFE). These membranes were utilized for the decomposition of Azitrox formulation, a powdered oral suspension that contains 200 mg of the antibiotic azithromycin per 5 mL. The degradation efficiency was monitored through chemical oxygen demand analysis. The most effective membrane (containing 2 wt.% TiO_2_-decorated GO) achieved an 80% degradation rate of azithromycin from the Azitrox formulation [245]. Zakeritabar et al. prepared PS-CeF_3_ membranes with different contents of CeF_3_. The membrane with the best photocatalytic activity under UV irradiation was 0.75% CeF_3_-PS. Membranes were used to treat pharmaceutical wastewater sourced from antibiotic producers’ effluents collected in Iran. The degradation efficiency was monitored through total organic carbon analysis. In that measurement, the best membrane 0.75% CeF_3_-PS showed 97% removal in COD measurement, which is a better result than a pristine membrane or other modified membranes [250].

## 13. Discussion

From the data in Table 4 can be concluded that pharmaceuticals are not the first choice of compounds to be removed when photocatalytic nanocomposite membranes are evaluated. Previous review articles about photocatalytic nanocomposite membranes are clear that dyes are the primary group of compounds on which photocatalytic nanocomposite membranes are tested [194,251,252]. The reason for that is the chemical structure of dyes, which is less complicated and easier to degrade in comparison to pharmaceuticals and pesticides. Another reason for that phenomenon is the cost of the research. Dyes can be detected, and their degradation or removal can be monitored by UV/Vis spectroscopy. Those instruments are far cheaper than HPLC instruments and much more than HPLC-MS instruments. When the degradation of pharmaceuticals is monitored via UV/Vis spectroscopy, information on transformation product formation during the process is lost. HPLC chromatograms, total organic carbon measurements and mass spectrometry spectra indicate the formation of transformation products. The formation of transformation products is one of the consequences of advanced oxidation processes for water treatment. In theory, with the use of advanced oxidation processes, like photocatalysis, pollutants should be mineralized to carbon dioxide and water. In real-world applications, complete mineralization is rarely achieved and pollutants are degraded into smaller molecules, which may or may not keep their harmful effect on humans and wildlife. After the photocatalytic treatment, toxicological tests should be used to estimate the efficiency of the photocatalytic process in the reduction in toxicity of the pollutants because transformation products can keep the activity from their parent compounds. There is only one example of testing after photocatalytic nanocomposite membrane treatment in this review. It can be argued that transformation products of pharmaceuticals would not pass the membrane during purification treatment with photocatalytic nanocomposite membranes, but that has to be examined.

Only four out of twenty-three published papers proposed degradation mechanisms with transformation products, and three of them were based on the results of other researchers. Those are the degradation mechanism of niflumic acid with a 10 wt.% TiO_2_-PVDF–TrFE nanocomposite membrane proposed by Aoudjit et al. [242], the mechanism for metronidazole degradation with a 10% Ag@TiO_2_/PVDF-HFP nanocomposite membrane proposed by Aoudjit et al. [244] and the degradation mechanism of norfloxacin with an Ag@TiO_2_/PVDF-HFP nanocomposite membrane proposed by Salazar et al. [248]. Singh et al. identified certain transformation products in the degradation mechanism of chloramphenicol with a Ca_10_(PO_4_)_6_(OH)_2_/TiO2-PSF nanocomposite membrane but did not propose a mechanism [249]. Chakachaka et al. proposed a degradation mechanism from their data obtained by LC-QTOF-MS analysis after the degradation of naproxen in water with a CoFe_2_O_4_-PES membrane. This is the only study out of 23 in which the proposed degradation mechanism was made from LC-MS data obtained in the survey. In this study, the degradation of naproxen goes through four pathways. The first, and dominant, is the attack of the hydroxyl radical on the naphthalene ring of naproxen, which leads to ring opening. In the second pathway, the superoxide radical attacks the central carbon atom in the propionic acid substituent of naproxen, causing decarboxylation. The third pathway occurs when a hydroxyl radical attacks the same carbon atom that has undergone decarboxylation causing hydroxylation, leading to redistribution and ring-opening reactions. The fourth pathway involves the reaction of *h*+ with the naphthalene ring, resulting in decarboxylation and ring opening. The products of these ring-opening reactions are lighter alcohols, which further react with radicals to be mineralized [228]. Those results are not surprising. Since membrane technologies are non-destructive methods of pollutant removal, scientists investigating them do not need expensive instruments that can monitor the formation of transformation products during pollutant degradation, such as coupled LC-MS systems.

The photocatalytic part of the photocatalytic nanocomposite membranes is usually a well-explored photocatalyst. In the case of this review, in 60% of the body of reviewed research, the photocatalysts of choice are TiO_2_ or TiO_2_-based photocatalysts. Examples of modifications of these photocatalysts are composites and doped variants. This is in line with the nature of that photocatalyst and its application serving as a nanocomposite in membranes. Due to the way photocatalytic membranes are composed, there is always a part of the photocatalyst that is embedded in the membrane or emerges into a layer of film that covers the membrane. This leads to a decrease in photocatalytic activity since only a part of the surface is exposed to illumination. When a photocatalyst is dispersed into the solution, it can react with its whole surface, but, being immobilised, only the exposed surface can react. That is the reason why modifications like cocatalysis and crystal facet engineering are not desired in this application because the cocatalyst or desired exposed facet might end up facing the membrane or a layer of the thin film in the coating. Changes in the morphology and crystal structure of TiO_2_ can be performed; however, P25 is the best-performing TiO_2_ photocatalyst in a field of research which has been ongoing for more than 50 years [253]. Constructing composites and doping are the two only plausible options. Doping is a modification of the crystal structure, and there is no fear of orientation of the photocatalyst in the membrane. The most used non-metal dopants for TiO_2_ are boron and nitrogen, while iron, platinum, zinc, gold and silver are among the most used metal dopants for TiO_2_ [254,255] TiO_2_ composites with WO_3_, Fe_2_O_3_, Bi_2_O_3_ or Cu_2_O can be synthesized in layers or stepwise, which helps prevent the inactivity of the photocatalyst. In the case of TiO_2_, using visible light increases its photocatalytic efficiency. This topic is the main point of other review papers [85,256].

Laboratory experiments with AOPs are usually performed on samples in ultrapure water, which is not the case in their wastewater application. In wastewater matrices undergoing tertiary treatment, there is organic matter, which can make layers on top of the membrane, causing fouling and deterioration of flux. Reactive oxygen species formed in AOPs can react with organic and inorganic matter in the water matrix and not with pollutants. Inorganic ions and anions can be drawn to the surface of the membrane and cause charge shielding and adsorption, changing the properties of the membrane in the process [231]. Inorganic ions can also act as scavengers for reactive oxygen species [233]. Those effects are also neglected in research, just like research with real wastewater matrices.

The list of individual pharmaceuticals in Table 4 is well-distributed. In seven studies (30% of the list), the photocatalytic nanocomposite membranes were tested on tetracycline, which makes tetracyline the most abundant pharmaceutical for the efficiency test of photocatalytic nanocomposite membranes. Tetracyclines are one of the most common families of antibiotics. In China, they are the most produced and used family of antibiotics. They ranked second worldwide in production and usage [257]. Ciprofloxacin [258], amoxicillin [259], azithromycin, diclofenac, 17β-estradiol and 17α-ethinylestradiol were at some point in time part of EU surface waters Watch List, which explains their popularity [260,261]. Others, except for ibuprofen, are less commonly used to test photocatalytic activity.

## 14. Conclusions

In general, the incorporation of nanoparticles into membrane structures has beneficial effects on the membrane, such as selectivity towards certain compounds and prolonged life span of a membrane. Those improvements can be hydrophilicity enhancement, which is manifested through higher permeability, higher rejection rate of specific compounds and antibacterial activity. Antifouling properties of nanocomposite photocatalytic membranes and improved chlorine resistance ensure the longevity of the membrane. The aim of new research should be to test photocatalytic nanocomposite membranes on substances which are common in wastewater. That means less dye research and more research on contaminants of emerging concern. Laboratory research should be conducted with real wastewater samples or secondary effluents. Those can also be matrices for pollutants. Interaction between pollutants, wastewater constituents and photocatalytic nanocomposite membranes should be more studied in order to achieve fouling mitigation and prolong the life span of the membrane. More research on new photocatalyst materials and their specific design for photocatalytic membranes is needed.

## Figures and Tables

**Table 1:** List of abbreviations.

Abbreviation	Name	Abbreviation	Name
ACA	Acetylacetone	PA	Polyamide
ACAiB	Methyl-2,4-heptanedione	PAA	Polyacrylic acid
ACA2iP	2,6-Dimethyl-3,5-heptanedione	PAN	Polyacrylonitrile
AMX	Amoxicillin	pDA (PDA)	Polydopamine
BSA	Bovine serum albumin	PES	Polyethersulfone
CA	Cellulose acetate	PTFE	Polytetrafluoroethylene
CQDs	Carbon quantum dots	PMIA	Poly(m-phenylene isophthalamide)
DCF	Diclofenac	PMMA	Polymethyl methacrylate
E2	17β-estradiol	PS	Polysulfone
EE2	17α-ethinylestradiol	PSF	Polysulfone
g-C_3_N_4_	Graphitic carbon nitride	PVC	Polyvinyl chloride
GO	Graphene oxide	PVDF	Polyvinylidene fluoride
HFP	Hexafluoropropylene	PVDF–TrFE	Polyvinylidene difluoride-co-trifluoroethylene
MWCNTs	Multiwalled carbon nanotubes	PVP	Polyvinylpyrrolidone
MF	Microfiltration	rGO	Reduced graphene oxide
MOFs	Metal–organic frameworks	RO	Reverse osmosis
NF	Nanofiltration	UF	Ultrafiltration

**Table 2:** Benefits of photocatalytic nanocomposite membranes without photocatalysis.

Membrane Process	Membrane	Nanoparticle	Effect	Ref.
NF	Si_3_N_4_/PAN	TiO_2_@rGO	enhancement in permeability and antifouling characteristics	[202]
UF	PVDF	ZnS/GO	enhanced permeability and antifouling performance	[203]
RO	PES and PA	TiO_2_	increase in permeance and salt rejection	[204]
RO	PA	TiO_2_	increase in water flux, fouling resistance and antibacterial activity	[205]
RO	PA	Titania nanosheet	improved surface hydrophilicity, surface roughness, improved working life of membrane	[206]
UF	PES/CA/PVP	TiO_2_	improvement in water permeability and hydrophilicity	[207]
RO	Al_2_O_3_ (α-alumina)	TiO_2_/ZrO_2_/ACA TiO_2_/ZrO_2_/ACAiB TiO_2_/ZrO_2_/ACA2iP	elevated selectivity at reduced pressure with comparatively low methanol flow rates	[208]
RO	PSF-PA	titanium dioxide/carbon dots (TiO_2_/CDs)	increase in desalination efficiency, permeability and antifouling properties, improved chlorine resistance	[209]
NF	PA	TiO_2_ and ZnO	improvement of fouling resistance and antibacterial properties	[210]

**Table 3:** Benefits of photocatalytic nanocomposite membranes with photocatalysis.

Membrane Process	Membrane	Nanoparticle	Pollutant	Effect	Reference
UF	PVDF	ZnS/GO	BSA	7.2% better separation	[203]
UF	PVDF	TiO_2_ and/or carbon nanotubes and/or BiVO_4_	BSA	differences between coated and incorporated materials	[215]
UF	PVDF	SnO_2_/GO	BSA	97.2% rejection rate of BSA under high flux	[216]
UF	PVC	TiO_2_/ZnO	humic acid	98.7% rejection rate of humic acid	[217]
UF	acrylic acid grafted microporous PVDF	TiO_2_, TiO_2_/GO	phenol	improvement of hydrophilicity, photocatalytic activity enhancement	[218]

**Table 4:** Pharmaceutical removal with photocatalytic nanocomposite membranes.

Pharmaceutical	Matrix Membrane	Photocatalyst	Concentration	Time (Light Exposure)	Photodegradation Rate	Reference
diclofenac	PMIA	WO_3_/g-C_3_N_4_	5 mg/L	150 min	85%	[227]
naproxen	PES	CoFe_2_O_4_	5 mg/L	180 min	60%	[228]
tetracycline	CA	Au_0.1_Ag_0.9_/TiO_2_	5 mg/L	120 min	90%	[229]
diclofenac	PSF	N,S-CQDs/TiO_2_	10 ppm	150 min	62,3%	[230]
diclofenac 17β-estradiol 17α-ethinylestradiol amoxicillin	α-Al_2_O_3_	Graphene/TiO_2_	500 μg/L (each, stock solution)	210 min	48%, 53% 56%, 65% 54%, 62% 49%, 45%	[231]
ciprofloxacin	PVDF/PMMA	TiO_2_	20 mg/L	30 min	15%	[232]
venlafaxine metoprolol	PVDF	TiO_2_	250 μg/L 250 μg/L	n.a.	99% 99%	[233]
tetracycline	PTFE	S-g-C_3_N_4_/N-TiO_2_	20 mg/L	120 min	99%	[234]
tetracycline	pDA/CA	Ti/BiOI	10 mg/L	120 min	98%	[235]
ciprofloxacin	PES	sulfonated graphene oxide/ZnO	10 mg/L	240 min	95.1%	[236]
tetracycline (hydrochloride)	graphene oxide	g-C_3_N_4_/TiO_2_	20 mg/L	240 min	98.77%	[237]
amoxicillin	PAA/PA	Ag-doped ZnO @Fe_3_O_4_/MWCNTs	50 ppm	120 min	60%	[238]
norfloxacin	PES	PDA-rGO/g-C_3_N_4_	10 mg/L	180 min	96.8%	[239]
tetracycline	PVDF	Au-TiO_2_	n.a.	120 min	75%	[240]
ciprofloxacin ibuprofen	PVDF–TrFE	TiO_2_	5 mg/L 15 mg/L	240 min 240 min	95% 48%	[241]
niflumic acid	PVDF–TrFE	TiO_2_	10 mg/L	360 min	91%	[242]
tetracycline	ZrO_2_	Mo-BiVO_4_	20 ppm	360 min	83%	[243]
metronidazole	PVDF-HFP	Ag@TiO_2_	10 mg/L	300 min	100%	[244]
azithromycin (Azitrox formulation)	PTFE	CA-GO-TiO_2_	200 mg azithromycin in 5 mL Azitrox formulation	120 min	80%	[245]
ibuprofen	PVDF	ZnO/Ag_2_CO_3_/Ag_2_O	20 mg/L	60 min	35.27%	[246]
tetracycline	PVDF	Bi_2_WO_6_/CeO_2_	20 mg/L	90 min	82%	[247]
norfloxacin	PVDF-HFP	Ag-TiO_2_	5 mg/L	300 min	80.7%	[248]
chloramphenicol	PSF	Ca_10_(PO_4_)_6_(OH)_2_/TiO_2_	50 mg/L	120 min	61.59%	[249]
pharmaceutical industry wastewater	PS	CeF_3_	n.a.	120 min	97%	[250]

## Data Availability

Not applicable.
